# Design and Simulation Analysis of a 3TnC MLC FeRAM Using a Nondestructive Readout and Offset-Canceled Sense Amplifier for High-Density Storage Applications

**DOI:** 10.3390/mi14081572

**Published:** 2023-08-09

**Authors:** Bo Peng, Donglin Zhang, Zhongqiang Wang, Jianguo Yang

**Affiliations:** 1 Key Laboratory of UV Light-Emitting Materials and Technology of Ministry of Education, Northeast Normal University, Changchun 130024, China; pengb806@nenu.edu.cn; 2 School of Microelectronics, University of Science and Technology of China, Hefei 230026, China; zhangdonglin20@mails.ucas.ac.cn; 3 Key Laboratory of Microelectronic Devices Integrated Technology, Institute of Microelectronics, Chinese Academy of Sciences, Beijing 100029, China; 4 Research Center for Intelligent Computing Hardware, Zhejiang Lab, Hangzhou 311121, China

**Keywords:** FeRAM, nondestructive readout, offset-canceled sense amplifier

## Abstract

Hf_0.5_Zr_0.5_O_2_-based multi-level cell (MLC) ferroelectric random-access memory (FeRAM) has great potential for high-density storage applications. However, it is usually limited by the issues of a small operation margin and a large input offset. The study of circuit design and optimization for MLC FeRAM is necessary to solve these problems. In this work, we propose and simulate a configuration for a Hf_0.5_Zr_0.5_O_2_-based 3TnC MLC FeRAM macro circuit, which also presents a high area efficiency of 12F^2^ for each bit. Eight polarization states can be distinguished in a single fabricated Hf_0.5_Zr_0.5_O_2_-based memory device for potential MLC application, which is also simulated by a SPICE model for the subsequent circuit design. Therein, a nondestructive readout approach is adopted to expand the reading margin to 450 mV between adjacent storage levels, while a capacitorless offset-canceled sense amplifier (SA) is designed to reduce the offset voltage to 20 mV, which improves the readout reliability of multi-level states. Finally, a 4 Mb MLC FeRAM macro is simulated and verified using a GSMC 130 nm CMOS process. This study provides the foundation of circuit design for the practical fabrication of a Hf_0.5_Zr_0.5_O_2_-based MLC FeRAM chip in the future, which also suggests its potential for high-density storage applications.

## 1. Introduction

Ferroelectric random-access memory (FeRAM) based on Hf_0.5_Zr_0.5_O_2_ film has attracted great attention because of its potential advantages of fast programming speeds [1,2], low operating power [3,4], and good CMOS compatibility [5,6]. Therefore, Hf_0.5_Zr_0.5_O_2_-based FeRAM is usually considered as one of the promising candidates for next-generation nonvolatile memory. In principle, Hf_0.5_Zr_0.5_O_2_-based FeRAM benefits from its thin thickness and its compatibility to the advanced CMOS process node [7]. It also can meet the requirements of today’s integrated circuits for high-density storage applications. However, the scaling of FeRAM capacitors is still limited compared to that of transistors, leading to low area efficiency. For instance, the ferroelectric capacitor (FeCAP) area was 40 × 10^3^ nm^2^ for the 28 nm node in Stefan et al.’s work [8], and the FeRAM area was 0.49 um^2^ for the 130 nm node in Zhao et al.’s work [9]. Considering the above reasons, multi-level cell (MLC) FeRAM for high-density storage applications has also been continuously explored in recent studies. For instance, K. Asari et al. used a three-pulse accessing scheme to achieve multi-level technology for FeRAM-embedded reconfigurable hardware [10]. Kai Ni et al. demonstrated one type of MLC non-volatile memory by fabricating three ferroelectric-insulator layer-based structures [11]. However, some problems still need to be solved before its practical application, such as the small operation margin of MLC FeRAM and the large input offset of a readout circuit SA. These issues usually lead to the misreading of memory states, thus restricting the application of MLC FeRAM to high-density storage. The circuit design is usually considered as one critical step to make the connection between the study of a single device and the practical fabrication of microchips. It can help to solve some problems that cannot be overcome in device studies and can be used in trial-and-error approaches before chip fabrication to save the economic cost and time cost. Therefore, it is necessary to solve the issues of a small operation margin and a large input offset of MLC FeRAM using the circuit design and optimization.

In this work, we propose a configuration of Hf_0.5_Zr_0.5_O_2_-based 3TnC MLC FeRAM with good area efficiency. The nondestructive readout approach is used, and a capacitorless offset-canceled SA is designed to solve the abovementioned issues, which leads to a wide operation margin and read reliability. The experimental electrical characteristics and a SPICE model of a Hf_0.5_Zr_0.5_O_2_-based FeRAM device are introduced first in this paper, which presents eight polarization states for MLC. Subsequently, the circuit structure and the operation of a 3TnC MLC FeRAM macro are presented in the following sections. Then, the capacitorless offset-canceled SA is proposed to minimize the mismatch of the readout transistor and the readout circuit. Finally, the layout of the 4 Mb 3TnC MLC FeRAM is provided with high area efficiency.

## 2. FeRAM Device Characteristics and SPICE Model

**Figure 1:**
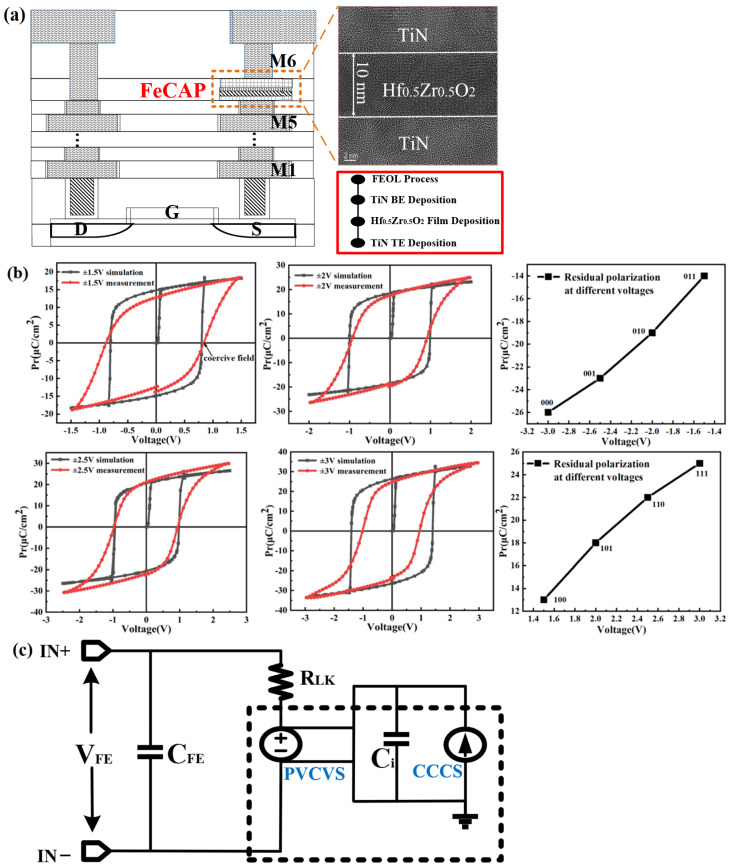
(**a**) Cross-sectional TEM image of the single Hf_0.5_Zr_0.5_O_2_-based FeCAP device; the inset shows the BEOL process of the FeCAP device; (**b**) measured and simulated polarization–voltage curve with different voltages of ±1.5 V, ±2 V, ±2.5 V, and ±3 V; (**c**) SPICE model of the FeRAM.

Figure 1a shows that the FeCAP cells are integrated between the metal layers of M5 and M6 in the GSMC 130 nm logic process. After the front-end-of-line (FEOL) process, the FeCAP device was fabricated by utilizing the back-end-of-line (BEOL) process [12,13], as shown in the bottom right inset of Figure 1a. Firstly, TiN film was deposited as the bottom electrode (BE) by using radio frequency (RF) reactive sputtering. Subsequently, the Hf_0.5_Zr_0.5_O_2_ film with a thickness of 10 nm was deposited on the BE via atomic layer deposition (ALD), in which the stoichiometric ratio of the Hf and Zr elements was 1:1. Finally, TiN was deposited as the top electrode (TE) via RF reactive sputtering, followed by a step of rapid thermal annealing. Through these above fabrication steps, we experimentally fabricated the Hf_0.5_Zr_0.5_O_2_-based FeCAP devices, and the size of each single device was 0.7 μm × 0.7 μm. The upper right inset of Figure 1a shows the transmission electron microscopy (TEM) image of a single Hf_0.5_Zr_0.5_O_2_-based FeCAP device, which shows its metal–ferroelectric–metal structure and confirms the 10 nm thickness of the Hf_0.5_Zr_0.5_O_2_ film. Figure 1b shows the experimentally measured P–V hysteresis curves and the simulated curve using a SPICE model, in which different sweep voltages of ±1.5 V, ±2.0 V, ±2.5 V, and ±3.0 V were utilized to perform the multiple level states. The P–V hysteresis curves were measured using a ferroelectric tester (Precision Premier II, Radiant Technologies, Inc., Albuquerque, NM, USA). Taking the sweep voltage of ±1.5 V as an example, the value of remnant polarization (Pr) was estimated to be 13 μC/cm^2^. It also can be seen that the remanent polarization became larger when increasing the applied voltages, while these different remanent polarization states represent its potential application for multiple-level storage. Figure 1b summarizes the eight positive and negative polarization states measured by using different applied voltages, which can be defined as the states from “111” to “000”. Thus, the eight polarization states can correspond to three bits in one single device of MLC FeRAM.

In order to make the subsequent circuit design of MLC FeRAM, stimulation was necessary to ensure a good fit with the experimental P–V curve, thus ensuring the simulation result was compatible with the performance of real devices. To simulate the electrical characteristics of our MLC FeRAM, we utilized the physics-based circuit-compatible SPICE model based on the single-domain approximation, referring to the literature by Aziz et al. [14], as shown in Figure 1c. In fact, this model specifically focuses on the compatibility of FEFET-based circuits with efficient design and analysis. This SPICE model is described using the time-dependent Landau–Khalatnikov equation [15] as follows:

(1) $$E-\frac{\rho dP}{dt}=\alpha P+\beta P^{3}+\gamma P^{5}$$

where *ρ* is the kinetic coefficient; *α*, *β*, and *γ* are the static parameters of the ferroelectric layer; *P* is the FeRAM remanent polarization; and *E* is the applied voltage on the FeRAM device. Further, by setting *Q_P_*, *T_FE_*, and *A_FE_* as the polarization charge stored in the FeRAM, the thickness, and the area of the FeRAM device, respectively, the time-dependent Landau–Khalatnikov equation can be described as follows:

(2) $$V_{FE}=V_{R_{LK}}+V_{C_{LK}}=\left(\rho \frac{T_{FE}}{A_{FE}}\frac{dQ_{P}}{dt}\right)+\left[T_{FE}\left(\frac{\alpha Q_{P}}{A_{FE}}+\frac{\beta {Q_{P}}^{3}}{A_{FE}^{3}}+\frac{\gamma {Q_{P}}^{5}}{A_{FE}^{5}}\right)\right]$$

FeRAM is modeled as a nonlinear capacitor (*C_LK_*) that is connected in series with a resistor (*R_L__K_* = *ρ* × *T_FE_*/*A_FE_*), in which the nonlinear capacitor is simplified to the polynomial voltage-controlled voltage source (PVCVS). As the current flows through *R_LK_* and PVCVS, the current is captured through the current-controlled current source (CCCS). Then, the *C_i_* of 1 *F* is charged by the current of CCCS, while the voltage across the CCCS is equal to the *Q_P_* in FeRAM. Therefore, the dashed block diagram implements the formula (*T_FE_* × (*αQ_P_*/*A_FE_* + *βQ*^3^*_P_*/*A*^3^*_FE_* + *γQ*^5^*_P_*/*A*^5^*_FE_*)). Finally, the voltage drop of FeRAM is equal to the sum of the voltage drop of the nonlinear capacitor *C_LK_* and resistor *R_LK_*, which implements the Landau–Khalatnikov equation. The P–V curve can be simulated by calculating the remanent polarization *P* = *Q_P_/A_FE_* and monitoring the applied voltage *V_FE_* on FeRAM. Table 1 summarizes the parameters used in this model for MLC FeRAM, in which C_FE_ is the parasitic parameter of FeRAM. By adjusting the values of PVCVS (*α*, *β*, *γ*) and the parasitic parameter, the *P*–*V* hysteresis curves of the MLC FeRAM device were simulated under different sweep voltages of ±1.5 V, ±2.0 V, ±2.5 V, and ±3.0 V, respectively. As shown in Figure 1b, the simulated P–V curves of this model can fit well with the experimentally measured data of the FeRAM device, which also ensures its feasibility in the subsequent circuit design. This model will be used for the design and simulation of a 3TnC MLC FeRAM macro circuit, as discussed in later sections.

## 3. Circuit Structure and Operation of 3TnC MLC FeRAM Macro

**Figure 2:**
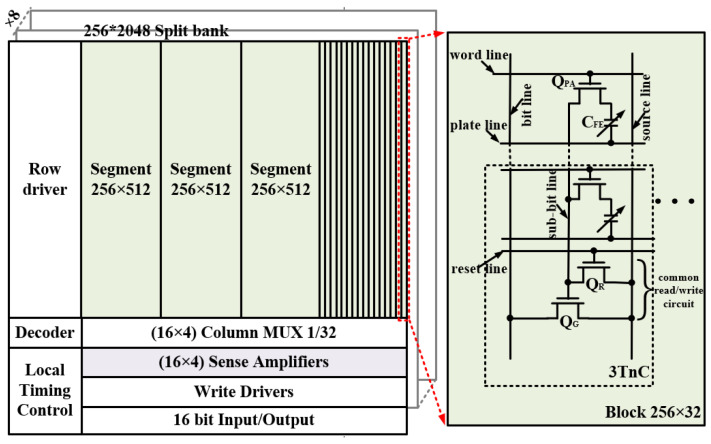
The 3TnC MLC array and peripheral circuit.

Figure 2 shows the circuit structure of our 4 Mb 3TnC MLC FeRAM macro, which comprises one 4 Mb bank and the peripheral circuit. The 4 Mb bank consists of eight 512 Kb split banks, while each split bank contains 256 word-lines (WL) or plate-lines (PL) and 2048 bit-lines (BL). Herein, one split bank includes four 128 Kb segments, where each segment contains 256 WLs or PLs and 512 BLs. Further, one segment includes sixteen 8 Kb blocks, where each block contains 256 WLs or PLs and 32 BLs and 32 3TnC arrays. The 3TnC means that there is one pass transistor *Q_PA_*, one reset transistor *Q_R_*, one pass transistor *Q_PA_*, and one MLC FeCAP in a minimum memory unit. Therein, a reset transistor *Q_R_* and a readout transistor *Q_G_*, as a common read/write circuit, are shared by 256 memory units in one array. Meanwhile, the pass transistor *Q_PA_* only has the switch function. Therefore, the 3TnC also means there are three types of transistors (*Q_R_*, *Q_G_* and *Q_PA_*) and 256 FeCAPs in one memory array. In the peripheral circuit, one 1/32 column mux corresponds to one block, while one split bank corresponds to a 16 × 4 column mux. Similarly, one split bank includes 16 × 4 sense amplifiers. The row driver and decoder are used to address and decode. The local timing control circuit can drive the pulse sequence of the write operation and nondestructive readout. Finally, by selecting one of eight split banks and four segments, the output of 16 bits is obtained for the MLC FeRAM macro.

To expand the reading margin between adjacent storage levels in MLC FeRAM, we used a nondestructive readout scheme. In the traditional 1T1C array, the destructive readout scheme usually adopts the operation mode of power supply voltage *V_DD_* to read out and write back. In comparison, the use of *V_RD_* (less than the coercive field voltage) in our nondestructive readout scheme did not destroy the residual polarization between the adjacent levels of MLC FeRAM, thereby avoiding the misreading of the stored data between adjacent levels. This scheme is beneficial for improving the read reliability characteristics of MLC FeRAM [16].

**Figure 3:**
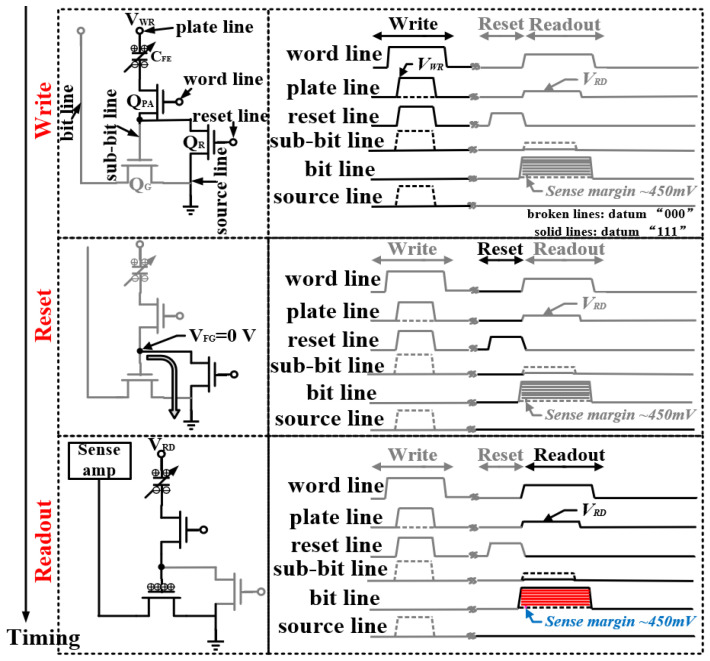
The principle of the nondestructive readout scheme and driving pulse sequence waveform.

An operation sequence for the nondestructive readout scheme is given according to Figure 3. Firstly, in the writing phase, a pass transistor *Q_PA_* and a reset transistor *Q_R_* turn on, which corresponds to the WL and the reset line (RL) turning on. Then, either the PL is applied with the write pulse *V_WR_* for the data “111”, or the source line is applied with the write pulse for the data “000”. Secondly, during the reset phase, a pass transistor *Q_PA_* turns off and a reset transistor *Q_R_* turns on, which corresponds to the WL turning off and the RL turning on. This step leads to removing the residual charge on the floating gate *Q_G_*. Finally, in the readout phase, a pass transistor *Q_PA_* turns on and a reset transistor *Q_R_* turns off, which means the WL is turned on and the RL is turned off. By applying the voltage V_RD_ (less than the coercive field voltage) to the PL, the FeRAM-stored data are read out to the BL through the readout transistor *Q_G_*. In the last step, since the readout scheme is nondestructive, the readout transistor *Q_G_*, as a gain cell, can expand the reading margin of FeRAM [17]. Therein, the sense margin can reach approximately 450 mV between two adjacent storage levels. This large sense margin can meet the requirement for distinguishing the eight different states from the “000” state to the “111” state for MLC FeRAM.

**Figure 4:**
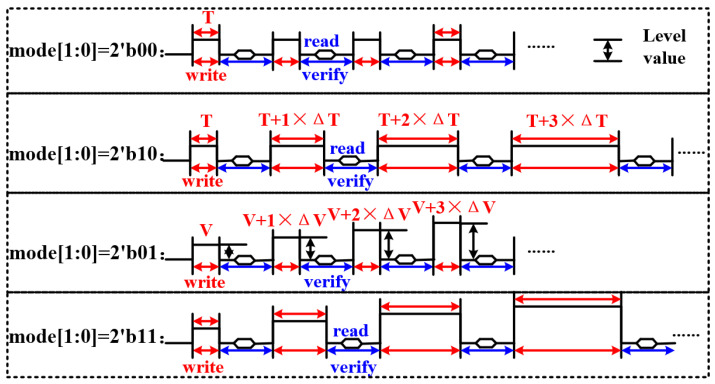
The overall pulse sequence of the write-verify scheme.

Figure 4 shows the overall pulse sequence diagram of the write–verify scheme. Due to the different residual polarization states of MLC FeRAM obtained by applying different voltages, the pulse sequence mode should be 2′b01 or 2′b11. However, to ensure the correctness of the written data for MLC FeRAM, the verify operation is added after the write operation, that is, the readout operation. If the read data are the same as the estimated data, which means the verification is correct, the pulse sequence continues to write the next adjacent storage level of the MLC FeRAM. If the verification is wrong, the pulse sequence mode enters 2b′00 or 2b′10 until the verification is correct. It should be emphasized that the polarization reversal of the ferroelectric domains is a relaxation phenomenon. Thus, the overall pulse sequence of different pulse widths is required to adjust the effectiveness of the written data for MLC FeRAM.

**Figure 5:**
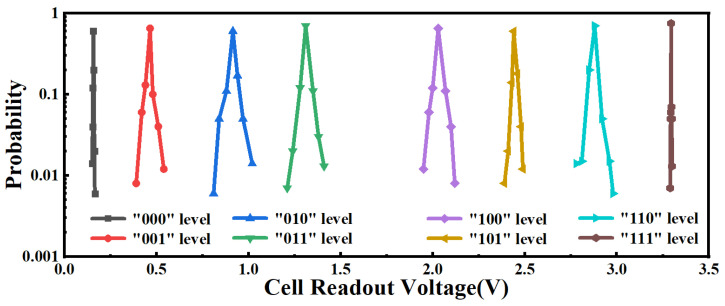
The readout voltage distribution diagram for each storage cell level based on the 16 Kb MLC array simulation.

Owing to the ideal electrical characteristics of the SPICE model of FeRAM, we adopt the 2′b01 mode to simulate the distribution condition of the readout voltage for each storage unit level of the MLC FeRAM. After 10 k Monte-Carlo simulations in the 16 Kb MLC array, each storage cell level can be effectively distinguished without the overlapping of the readout voltage distribution, as shown in Figure 5. At the same time, it can be seen that there is a nearly 450 mV reading margin between each storage unit level of MLC FeRAM.

## 4. Capacitorless Offset-Canceled Sense Amplifier

Due to the fluctuation of the CMOS process, there is usually a mismatch phenomenon in the readout transistor *Q_G_* of the 3TnC cell array and the readout circuit SA, resulting in a large input offset. To improve the reliability of the readout stored data between adjacent storage levels in MLC FeRAM, we propose a capacitorless offset-canceled SA to minimize the mismatch of SA and readout transistor. Meanwhile, compared with the single-capacitor offset-canceled SA [18], the capacitorless offset-canceled SA uses the parasitic capacitor of a transistor to replace the original single metal/insulator/metal (MIM) capacitor, thus saving the area of whole chip.

**Figure 6:**
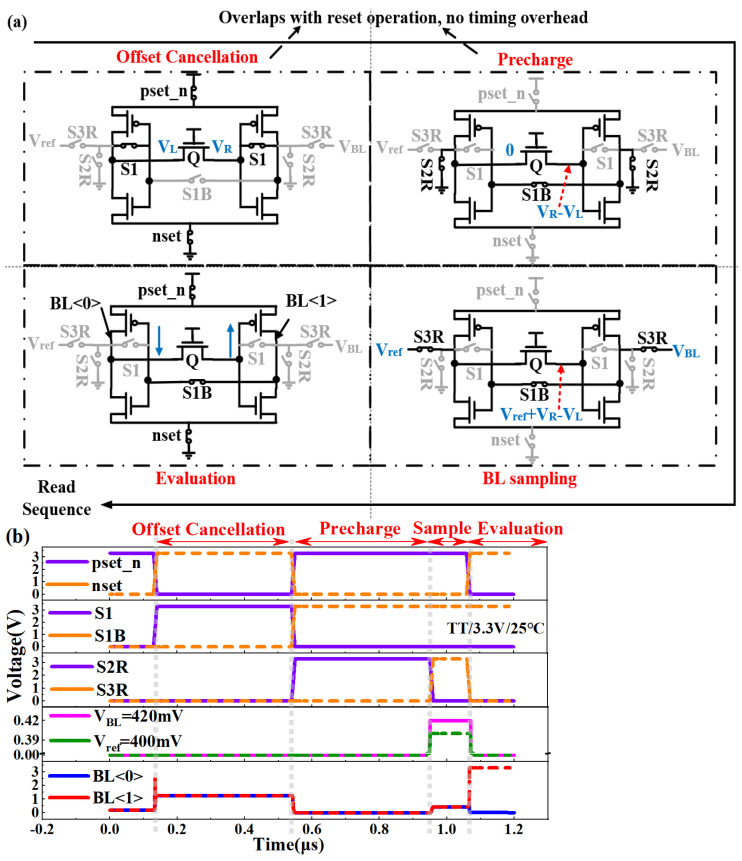
(**a**) The operation principle of the capacitorless offset-canceled SA; (**b**) the simulated waveform of the proposed SA.

The minimization mismatch principle of capacitorless offset-canceled SA is explained below. Firstly, in the offset cancellation phase, the outputs of inverters are connected to their inputs in Figure 6a, which correspondingly close the switches of “pset_n”, “nset”, and “S1” in Figure 6b. The parasitic capacitor of transistor *Q* collects the trip voltage of inverters, leading to the formation of two voltages of *V_L_* and *V_R_* at the two sides of transistor *Q*. Secondly, in the precharge phase, one side of the parasitic capacitor of transistor *Q* is connected to the ground, which correspondingly closes the switches of “S2R” and “S1B”, while keeping the other switches open. Therefore, the other side of the parasitic capacitor of transistor *Q* obtains the voltage *V_R_* − *V_L_*, which is the difference between the two trip voltages of *V_L_* and *V_R_*. In the BL sampling phase, the switches of “S3R” and “S1B” are closed, while the other switches are open. The different reference voltage *V_ref_* is added to the voltage *V_R_* − *V_L_* for different storage levels of MLC FeRAM, which compensates for the mismatch of the two side inverters, thus canceling out the offset of the SA. Finally, in the evaluation phase, the switches of “pset_n”, “nset”, and “S1B” are closed, while the other switches are open. The SA can be quickly sensed thanks to the canceling out of this offset. Under the conditions of a TT process corner, 3.3 V, and 25 °C, Figure 6b shows the simulation result of the output waveforms of “BL<0>” and “BL<1>” in the SA. Herein, it is noted that the offset cancellation and precharge phases can be run concurrently with the reset operation of the 3TnC array, thus avoiding any timing penalty for the proposed method.

**Figure 7:**
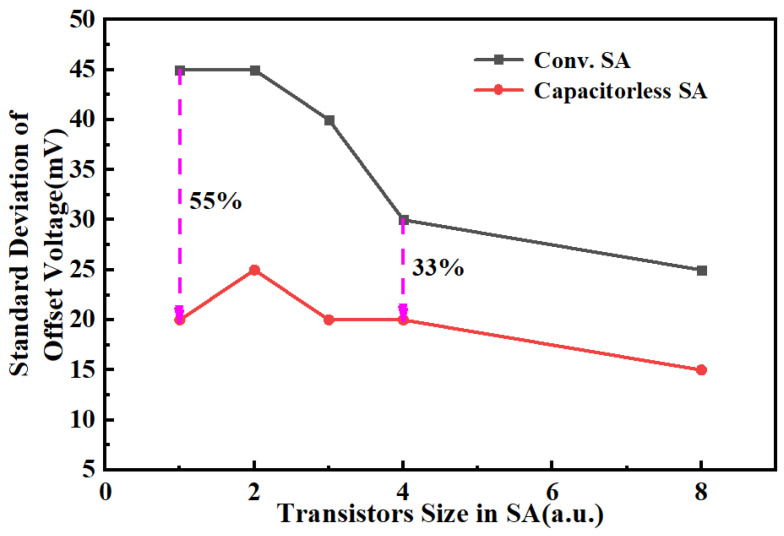
The comparison of the offset voltage of capacitorless SA and conventional SA.

Figure 7 shows the relationship between the input offset voltage and transistor size for both the proposed SA (capacitorless SA) and the conventional SA (conv. SA). Generally, the mismatch of transistors in the SA minimizes with the increment in its size, which means the input offset of all transistors of the SA also reduces accordingly. Importantly, after 10 k Monte-Carlo simulations, compared to the conv. SA, the standard deviation of the input offset can be reduced on average by nearly 45% in the proposed SA due to its minimization mismatch principle. Meanwhile, compared to the single MIM-capacitor SA with the same offset voltage and CMOS process, the area of capacitorless SA can be decreased by 35%.

## 5. The Layout of 4 Mb 3TnC MLC FeRAM and a Comparison with Other Memory Works

**Figure 8:**
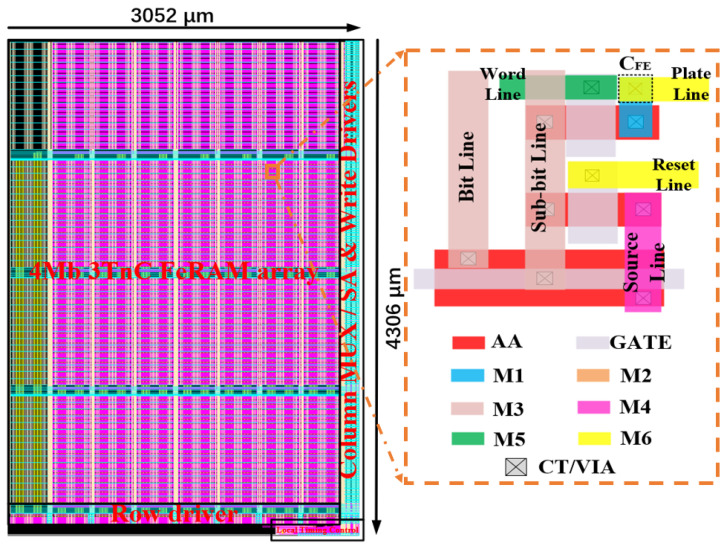
The layout of 4 Mb 3TnC MLC FeRAM; the inset shows the layout of a 3T1C cell array.

Figure 8 shows the layout of 4 Mb 3TnC MLC FeRAM, with an area of 3052 × 4306 μm^2^, consisting of the 3TnC cell array, the capacitorless SA, and the other peripheral circuits. The inset shows the layout of the single 3T1C cell array. “AA” and “GATE” mean the active area and gate electrode of transistor. Table 2 illustrates the performance comparison of our work with other memory works. The proposed 3TnC MLC FeRAM macro has the advantages of the high area efficiency of 12F^2^ for each bit, a large sense margin of 450 mV between each level of storage data, and a low offset of 20 mV, which are all beneficial for high-density storage applications. Both the read and write time of the cell are 100 ns, while the max power consumption is 48.4 μW for a read and a write operation. Here, it should be noted that the influence of temperature on FeRAM is relatively small, as reported in reference [19]; the variation in the readout voltage of FeRAM is about 50 mV; and the readout margin of adjacent polarized states of our FeRAM with 3TnC architecture is 450 mV. Therefore, our 3TnC MLC FeRAM chip has good stability.

## 6. Conclusions

In this work, a novel 3TnC MLC Hf_0.5_Zr_0.5_O_2_-based FeRAM with a high area efficiency of 12F^2^ for each bit is proposed for high-density storage application. Eight polarization states (three bits) can be obtained in one MLC FeRAM. The corresponding timing operation using a nondestructive readout is verified via simulation based on the GSMC 130 nm CMOS process. Meanwhile, the readout circuit SA has a low offset of 20 mV and a large sense margin of 450 mV to improve the reliability of the reading of the stored data between each level of the MLC FeRAM. These advantages of 3TnC MLC FeRAM using nondestructive readout and capacitorless SA ensure its potential for future high-density storage applications.

## Figures and Tables

**Table 1:** The spice model parameters of MLC FeRAM.

Model Parameter	*α* (m/F)	*β* (m^5^/F/C^2^)	*γ* (m^9^/F/C^4^)	*R_LK_* (Ω)	*C_FE_* (F)
±1.5 V simulation	−2.25 × 10^13^	3.06 × 10^39^	2.3 × 10^65^	1.0 M	1.0 f
±2 V simulation	−2.25 × 10^13^	2.06 × 10^39^	8.2 × 10^64^	0.9 M	1.0 f
±2.5 V simulation	−2.24 × 10^13^	1.62 × 10^39^	2.6 × 10^64^	0.8 M	0.9 f
±3 V simulation	−2.23 × 10^13^	1.02 × 10^39^	1.9 × 10^64^	0.75 M	0.85 f

**Table 2:** The performance compared to other memory works.

	This Work	Ref [8]	Ref [9]	Ref [19]
Cell structure	3TnC	2TnC	1T1C	1T1C
Technology	130 nm	130 nm	130 nm	130 nm
Multi-level cell	Yes	Yes	No	No
Area (F^2^/bit)	12	51	36	36
SA offset	20 mV	N/A	45 mV	18.1 mV
Max sense margin	450 mV	300 mV	270 mV	200 mV
Read time	100 ns	15 μs	150 ns	5 ns
Write time	100 ns	15 μs	150 ns	7 ns
Power consumption	48.4 μW	18 μW	N/A	N/A

## Data Availability

The data that support the findings of this study are available from the corresponding author upon request.
